# Eco-Friendly Analytical Approach for Sensitive Spectrofluorimetric Determination of the Flavonoid Chrysin in Capsules and Human Plasma

**DOI:** 10.1007/s10895-024-03962-9

**Published:** 2024-10-24

**Authors:** Heba Abd El-Aziz, Nada E. Hammouda, Fathallah Belal, Heba Samir Elama

**Affiliations:** 1 Pharmaceutical Analytical Chemistry Department, Faculty of Pharmacy, Mansoura University, Mansoura, 35516 Egypt; 2 Unit of Drug Analysis, Faculty of Pharmacy, Mansoura University, Mansoura, 35516 Egypt; 3 Department of Pharmaceutical Analytical Chemistry, Faculty of Pharmacy, Mansoura National University, Gamasa, 7731168 Egypt

**Keywords:** Chrysin, Flavonoid, Fluorescence, Analysis, Human plasma

## Abstract

Chrysin is a plant flavonoid that has different therapeutic effects as anti-inflammatory, anti-cancer, anti-oxidant, and immune booster. Spectrofluorimetry has received a lot of interest lately because of its ecological greenness and analytical performance. This approach employed the native fluorescence of chrysin at 339 nm following excitation at 231 nm in distilled water. Modern advances in analytical chemistry have been used to lessen occupational and environmental concerns by employing distilled water as a dilution solvent through method development and application. The approach was found to be excellent green supported by eco-scale score of 97 and 0.94 AGREE rating, in addition to an overall whiteness score of 88.80. The design aimed to analyze chrysin in raw materials, Chrysin® capsules and human plasma. The method was linear over 0.5–7.0 ng mL^⁻1^ chrysin, with LOD of 0.06 ng mL^⁻1^ and LOQ of 0.20 ng mL^⁻1^. The offered method was effectively applied for determination of chrysin in the commercial capsules Chrysin® and spiked human plasma samples with average recoveries of 99.76% and 99.98%, respectively for capsules and spiked human plasma. Up to date, no spectrofluorimetric method has been described for chrysin analysis, then, this presented an opportunity to develop a sensitive, quick, reliable, environmentally friendly, and valid fluorescence-based method.

## Introduction

**Fig. 1:**
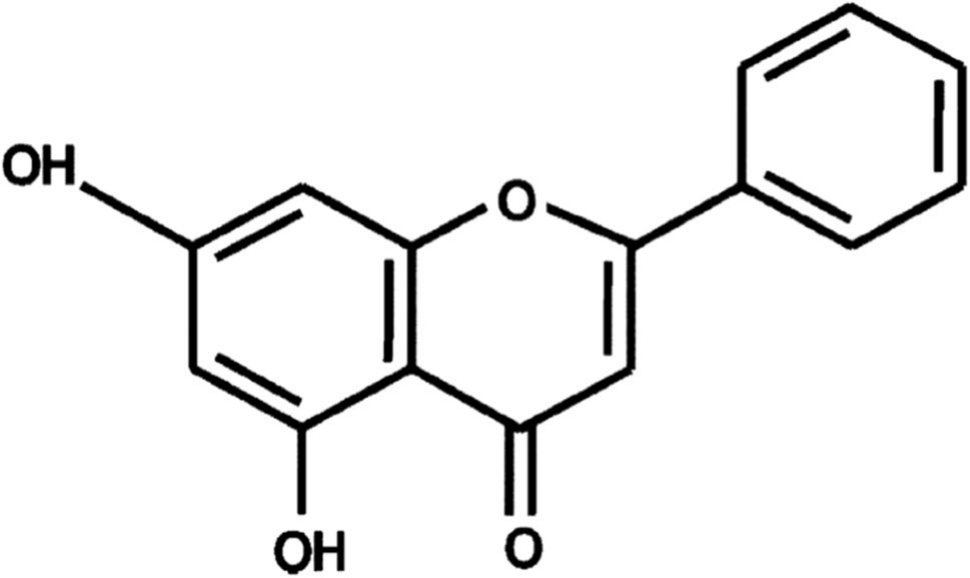
Structural formula of Chrysin

The interest in plant flavonoids and their potential health benefits has significantly risen in recent times, prompting many studies [1]. The focus of this study is mainly on chrysin (CRS), a type of flavone (5,7-dihydroxyflavone) as shown in Fig. 1, that is naturally present in multiple plants [2]. CRS, a type of flavonoid compound, is present in various natural sources such as honey, propolis, and passionflower. Recently, it has been the centre of attention for numerous studies due to its possible therapeutic effects in several conditions [3].

CRS possesses tremendous potential therapeutic effects as an anti-inflammatory, this property may be beneficial in reducing inflammation-related disorders such as arthritis and allergies [4]. Also, anti-oxidant effects that may help decrease oxidative stress and damage, which is caused by free radicals [5]. A cardiovascular and metabolic effects: CRS has been shown to lower blood pressure and improve glucose and lipid metabolism [5]. Furthermore, anti-cancer effects: researchers have found that CRS can induce apoptosis in cancer cells, inhibit cancer cell proliferation, and block tumour growth [6]. CRS's ability to modulate the immune system has the potential to reduce the severity of autoimmune diseases [7].

In addition to its potential therapeutic effects, CRS may have applications in analytical methods. The utilization of flavonoid complexes as fluorescent probes is extensive in the detection of cations and biological proteins [8]. This is due to their environmentally friendly nature and low toxicity, which makes them an attractive option for researchers [9–11]. CRS is known to be a potent antioxidant, making it suitable for use in the development of sensors that can detect toxic heavy metals and environmental pollutants[12]. CRS have been studied for its effect on enzyme activity in both serum and urine [13–15].

In recent years, several analytical techniques have been employed for the determination of CRS, including UV–Vis spectrophotometry [16], RP HPLC [17–19], HPTLC [20], and LC–MS/MS [21]. However, these methods have limitations such as low sensitivity and resolution in spectrophotometry [16], prolonged analytical time in HPLC [17–19] and HPTLC [20], high cost in LC–MS/MS [21].

Since none of the reported approaches employed CRS's natural fluorescence properties. This study sought to develop a new quick, selective, and sensitive spectrofluorimetric method for assessing CRS in biological samples and pharmaceuticals.

Recent green chemistry developments are guided by 12 principles and aim to achieve both environmental and economic goals [22]. This work aimed to accomplish green chemistry aims, several green-assessment tools were applied to assess greenness of the method such as the analytical Eco-Scale [23], the green analytical procedure index (GAPI) [24], and the analytical greenness metric approach (AGREE) [25]. In addition, the method's balance and durability were evaluated using the most recent whiteness metric approach [26].

## Experimental

### Instruments

At voltage of 800 mV, Agilent® Cary Eclipse spectrofluorometer with a flash xenon lamp was operated for measurements. A smoothing factor of 20 and slit width of 5 nm were applied. High Performance Quartz Glass Hellma®, pathlength 10x10 mm, spectral range 200-2,500 nm, were used for measurements.

pH-Meter (Consort, P-901) was utilized to adjust buffer solutions pHs.

Vortex Mixer (IVM-300P, Taiwan) and Centrifuge (model 2-16P, SIGMA Laborzentrifugen, Germany) was used for separation of biological matrices. Wisd Wise Clean WUC A22H Ultrasonic Bath (Witeg, Germany) was used for samples sonication.

### Reagents and Materials

Researchers purchased analytical grade CRS with CAS number c80105-25G of 97 % purity and methanol (Fischer®) from Sigma Aldrich, Darmstadt, Germany.

Chrysin® capsules CA 92056 USA, manufactured for MRM®, containing 500 mg CRS per capsule, were used for method application.

From Sigma-Aldrich, Cairo, Egypt, analytical grades of cetrimide, beta cyclodextrin (βCD), tween 80, sodium dodecyl sulphate, and HPLC grade acetonitrile, ethyl acetate, ethanol, n-propanol, and methanol, were all acquired. Hospitals of Mansoura University in Dakahlia, Egypt donated the frozen plasma of a healthy 26-year-old donor, which was kept at -5˚ C until it was required.

Distilled water was obtained from faculty of pharmacy, Mansoura University.

### Standard Solutions

After precisely weighing 10.0 mg of CRS raw material, it was quantitatively transferred to a 100 mL volumetric flask to be dissolved in methanol. 100 µg. mL^⁻1^ was the ultimate standard concentration obtained. The stock solution was stable over two weeks when refrigerated. 0.2 mL from the prepared standard was transferred to another 100.0 mL volumetric flask, completed to the mark using distilled water to obtain the working standard, 200.0 ng mL^⁻1^.

### Procedures

#### CRS Calibration Graph

A set of 10.0 mL volumetric flasks was arranged, into which aliquots of CRS working standard were put so that the final diluted concentrations in Table 2 were attained upon dilution. Subsequently, double-distilled water was added to the flasks to the final level and carefully mixed. Relative fluorescence intensity (RFI) was observed at 339 nm following excitation at 231 nm against distilled water blank at room temperature. Calibration graph was obtained by plotting relative fluorescence intensity (RFI) data against the final concentration of CRS in ng mL^⁻1^. Instead, the matching linear regression equation was derived.

#### Applications

##### Average Content Determination of CRS in Chrysin® Capsules

**Chrysin**^®^ capsules are labelled to contain 500 mg CRS/ hard gelatine capsule. Ten **Chrysin**^®^ capsules were weighed, carefully emptied into a beaker, and the empty shells were reweighed and subtracted from the total weight. A weight equivalent to 10.0 mg CRS was transferred into 100.0 mL volumetric flask. After adding roughly 50.0 mL of methanol to the flask, it was sonicated for 30 minutes to ensure complete extraction of CRS, then the methanol was added all the way till the mark. After that, the solution in the flask was filtered, with the first ten millilitres of filtrate being rejected. Distilled water was used to produce additional dilutions in order to create working standard solution (200.0 ng mL^-1^) that would be examined using the methodology provided in subsection "2.4.1.". Using the calibration graph that had already been generated or the related regression equation, the average nominal content was determined to be 498.8 ± 6.6 mg.

##### Determination of CRS in Spiked Human Plasma Samples

Gentle thawing of the given frozen plasma was performed. A set of 15.0-mL centrifugation tubes were supplied with 1.0 mL aliquots of human plasma, which were then spiked with aliquots of CRS to reach the final studied concentrations in the range of 3.0–7.0 ng mL^⁻1^. The tubes were vortexed, and 2.0 mL of ethyl acetate were added to extract the CRS from the aqueous layer. Centrifugation was set to 5000 rpm for 10 min. one mL aliquot of the ethyl acetate layers evaporated at room temperature overnight. The remaining leftovers were dissolved in 1.0 mL of acetonitrile and quantitatively transferred to 5.0 mL volumetric flasks using distilled water as diluting solvent. Then optimal study procedure outlined in "2.4.1" was followed. The average plasma recovery was 99.98%.

## Results and Discussion

### Method Development

**Fig. 2:**
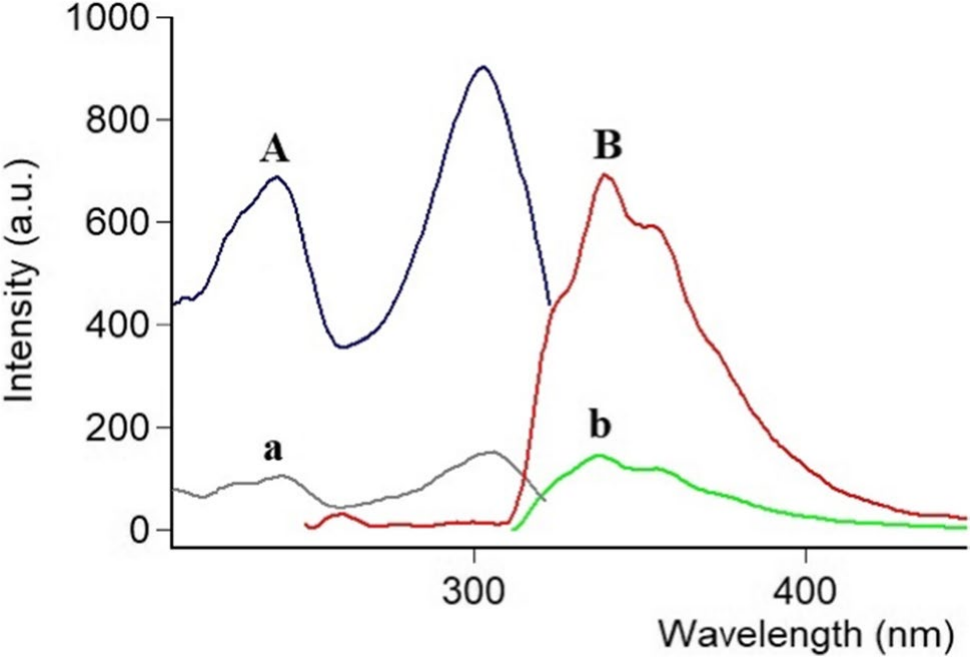
Fluorescence spectra: excitation and emission spectra of (**A** and **B**) 5.0 ng mL^⁻1^ CRS, while (a and b) blank: distilled water

Spectrofluorimetry as a technique can be utilized for identifying a wide range of chemicals in biological samples because of its high sensitivity (ng mL^⁻1^) and maximized selectivity, however, the intensity of the fluorescence reaction depends on several factors. The analyte could be determined utilizing its natural fluorescence that is structurally related, otherwise, a derivatization reaction is performed to convert it to a corresponding fluorophore [27]. CRS has a remarkable native fluorescence because it is characterized by a core conjugated 5,7-dihydroxyflavone ring to which a benzene ring is connected (Fig. 1). Excitation at 231 nm resulted in emission at 339 nm, as seen in the mirror image Fig. 2, which was obtained by scanning the CRS for excitation and emission wavelengths.

Furthermore, water was used as a diluting solvent, reinforcing economically friendly chemical recommendations that have been widely implemented to reduce environmental and occupational concerns as compared to organic solvents.

Factors affecting CRS’s RFI, such as pH, surfactant type, and diluting solvent, were thoroughly examined in order to obtain optimal sensitivity and linearity while maintaining maximum stability. Each element was carefully examined, while other factors were held constant using the univariate optimization technique.

#### Different pH Values’ Effect on RFI of CRS

The pH has a significant effect on the fluorescence spectrum, which can either increase or decrease RFI. This is primarily dependent on the analyte's chemical structure and if a specific pH boosts its resonance forms [28].

**Fig. 3:**
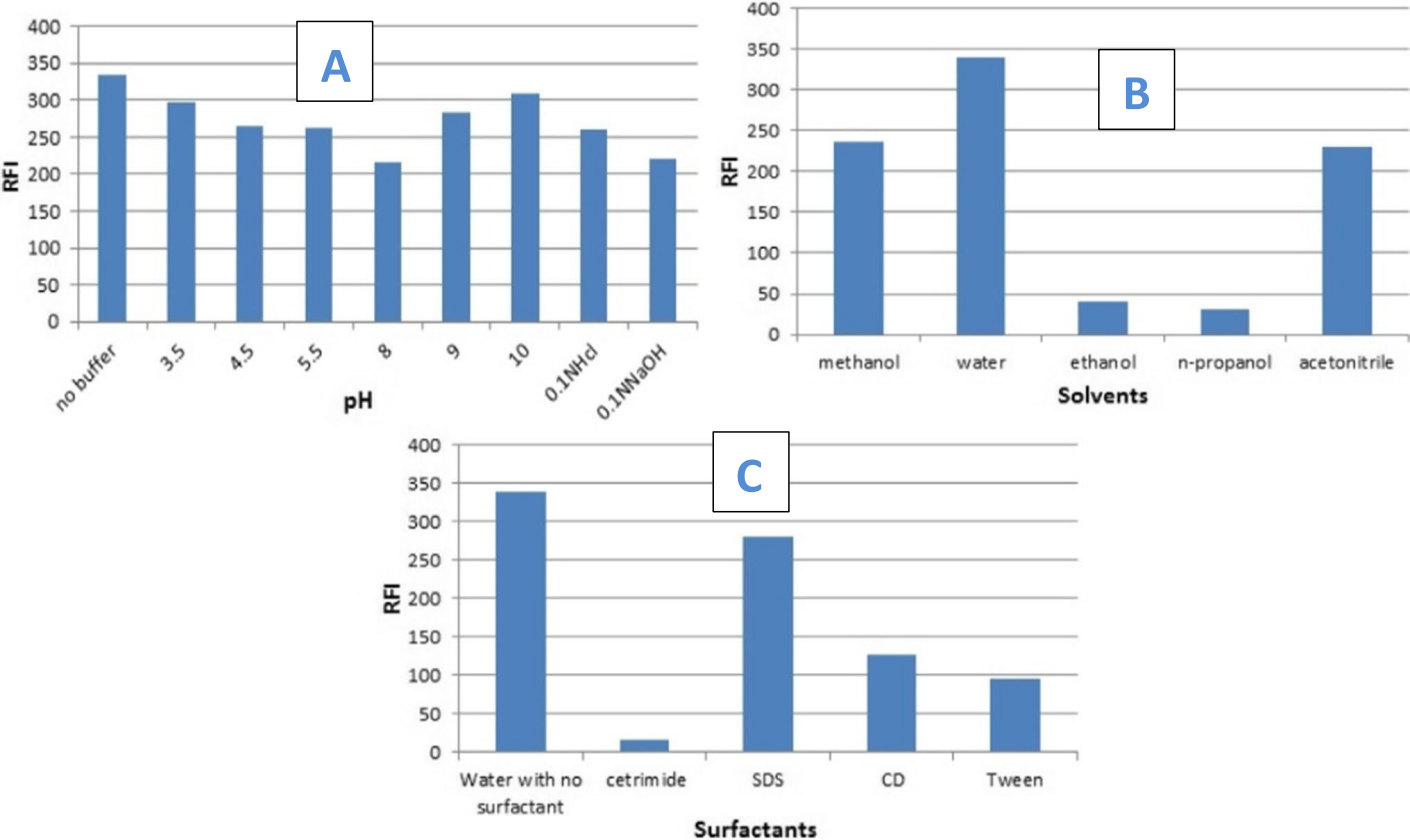
Effect of different (**a**) pH, (**b**) diluting solvents, and (**c**): surfactants, on the fluorescence intensity of 3.0 ng mL^⁻1^ CRS. All measurements were performed at 339 nm following excitation at 231 nm

As a result, multiple pH values ranging from 3.5 to 10.0 were tested using 0.1M Britton Robinson buffer. As indicated in Fig. 3, CRS's RFI was not significantly increased over the pH range examined. Furthermore, strong basic and acidic solutions (0.1 M NaOH and 0.1 M HCl) were examined, both downsized CRS's RFI. As a result, the study was proceeded without a buffering system.

#### Solvents’ Effect on RFI of CRS

A hydrogen-bonding solvent can affect the value of λ_max_ in excitation and emission spectra by modifying the energy levels of non-bonding electrons and electrons in π* orbitals [28]. Hence, a number of solvents were tested, including methanol, distilled water, ethanol, n-propanol, and acetonitrile. Using distilled water resulted in the highest achievable RFI for CRS. (Fig. 3).

#### Surfactants’ Effect on RFI of CRS

Any systematic change that affects the number or strength of collisions in the solution may influence the magnitude of RFI. Collisions enhance radiation less decay and the loss of excess energy as heat, therefore more or more violent collisions contribute to radiation less decay and diminish fluorescence emission [28]. The solvent viscosity will affect the number of collisions. Hence, practical investigations to promote solution viscosity were undertaken employing diverse surfactants including β-cyclodextrin, cetrimide, and sodium dodecyl sulfate (SDS), and tween 80, 1 gm% for each, which is above the critical micelle concentrations. The obtained results are depicted in Fig. 3 and shows that cetrimide, tween 80, and β-cyclodextrin conquered CRS's RFI, while SDS showed a relatively higher RFI but still lower than that obtained in distilled water without using surfactants. The net result suggested continuing the method without adding surfactants.

### Analytical Methodology Validation

#### Linearity and Range

The present method was validated in compliance with the FDA standards. [29].

**Fig. 4:**
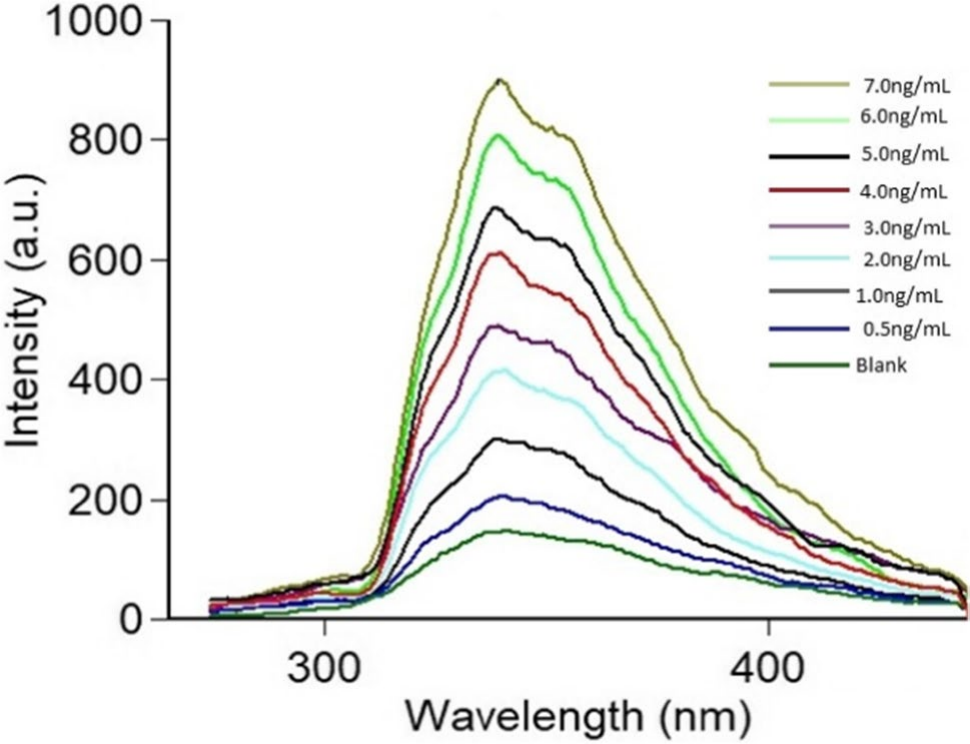
Fluorescence emission spectra of increasing concentration of CRS ranging from (0.5-7.0 ng mL^-1^) in presence of blank: distilled water. All measurements were performed at 339 nm following excitation at 231 nm

The CRS calibration curve was developed using eight distinct concentrations in the linearity range of 0.5 – 7.0 ng mL^⁻1^ by graphing the matching RFI against each concentration (Fig. 4). The range and regression equation were calculated as shown in Table 1.

**Table 1:** Analytical performance data for determination of CRS using the suggested spectrofluorimetric method

Validation parameter	Spectrofluorimetric method
λ_excitation/emision_ (nm)	231/339
Linearity range	0.5–7 ng mL^-1^
Intercept (a)	31.69
Slope (b)	103.66
Correlation coefficient (r)	0.9999
S.D. of residuals (S_y/x_)	3.01
S.D. of intercept (S_a_)	2.02
S.D. of slope (S_b_)	0.48
Limit of detection, LOD	0.06 ng mL^-1^
Limit of quantitation, LOQ	0.20 ng mL^-1^

#### Detection and Quantitation Limits

CRS had low limits of detection (LOD) and quantitation (LOQ) values of 0.06 and 0.20 ng mL^⁻1^ (Table 1), reflecting technique sensitivity. They were calculated using the two reported equations LOD = 3.3 S_a_/slope, and LOQ = 10 S_a_/slope, where S_a_; standard deviation of intercept.

#### Accuracy and Precision

To evaluate accuracy and precision, Our technique was compared to the comparative method [16], and our procedure's accuracy is stated in Table 2 and can be reflected by the acceptable values of student *t*-test and variance ratio F-test [30]. Table 2 shows the generated data and values of statistical analysis.

**Table 2:** Application of the suggested spectrofluorimetric method to the determination of CRS in its raw material

Parameters	Suggested spectrofluorimetric method				Reference method [16]
	Aliquots volume (mL)	Conc. taken (ng mL^−1^)	Conc. found (ng mL^−1^)	%Found^a^	% Found^a^
	0.025	0.50	0.49	98.02	101.91
	0.05	1.00	1.02	101.50	99.81
	0.10	2.00	2.01	100.47	98.95
	0.15	3.00	2.97	99.14	100.44
	0.20	4.00	4.04	100.88	
	0.25	5.00	4.95	99.03	
	0.30	6.00	6.02	100.29	
	0.35	7.00	7.01	100.09	
Mean ± S.D	99.94 ± 1.14				100.28 ± 1.25
Accuracy	Mean recovery% ± Er% 99.94 ± 0.50				
Student t test	0.49(2.23)*				
F- test	1.23 (4.35)*				

^a^Average of 3 replicate determinations^*^The values between parentheses are the tabulated values of *t* and *F* at *P* = 0.05 [30]. Reference method was based on direct spectrophotometric measurements of CRS at 367 nm in methanol [16]

Table 3 summarizes the suggested method's intra and inter-day precision results. Low percentage RSD and percent error values indicated reasonable intra- and inter-day precision. The acquired percentage recoveries and standard deviations reflect the procedure's excellent level of accuracy across multiple samples.

**Table 3:** Precision data for the determination of CRS applying the suggested spectrofluorimetric method

Concentration (ng mL^−1^)	Intra-day precision			Inter-day precision		
	Mean ± SD	RSD (%)	%Error	Mean ± SD	RSD (%)	%Error
1.0	101.09 ± 1.16	1.15	0.66	101.50 ± 1.20	1.19	0.68
3.0	99.14 ± 0.97	0.97	0.56	98.88 ± 1.07	1.07	0.62
6.0	100.31 ± 0.89	0.89	0.51	100.18 ± 1.22	1.22	0.70

### Applications

The average content of CRS was detected in the Chrysin^®^ oral capsule. The labeled CRS content was 500 mg each capsule. The proportion of CRS determined using the proposed approach was compared to the comparative spectrophotometric method using the Student's *t*-test and the F-test. The results in Table 4 show no noticeable differences between the two approaches regarding accuracy and precision.

**Table 4:** Application of the suggested spectrofluorimetric method to the determination of CRS in capsules

Pharmaceutical dosage forms	Proposed spectrofluorimetric Method		Reference Method [16]
	Conc. taken (ng mL^-1^)	% Found ^a^	% Found ^a^
Chrysin® capsule (500 mg CRS)	1.00	98.33	98.79
	3.00	101.22	102.14
	4.00	100.49	98.4
	6.00	99.00	100.62
Mean ± S.D.		99.76 ± 1.32	99.99 ± 1.73
% RSD		1.32	1.73
*Student t test*	0.21 (2.45)*		
F- test	1.70 (9.28)*		

^a^Average of 3 replicate determinations

*The values between parentheses are the tabulated values of *t* and *F* at *P* = 0.05 [30]

A metabolism study of CRS showed that plasma concentrations vary from 3.0 to 16.0 ng mL^⁻1^ [31]. The proposed methodology for determining CRS in human plasma applied liquid-liquid extraction. The organic solvent used was ethyl acetate because of good solubility of CRS. It was carefully selected to ensure extraction of CRS into the organic layer that was later subjected to evaporation. Ethyl acetate lies in third place after dimethyl formamide and acetone as solvents for CRS. It was chosen due to its adequate solubility of CRS alongside immiscibility with the aqueous layer [32].

**Fig. 5:**
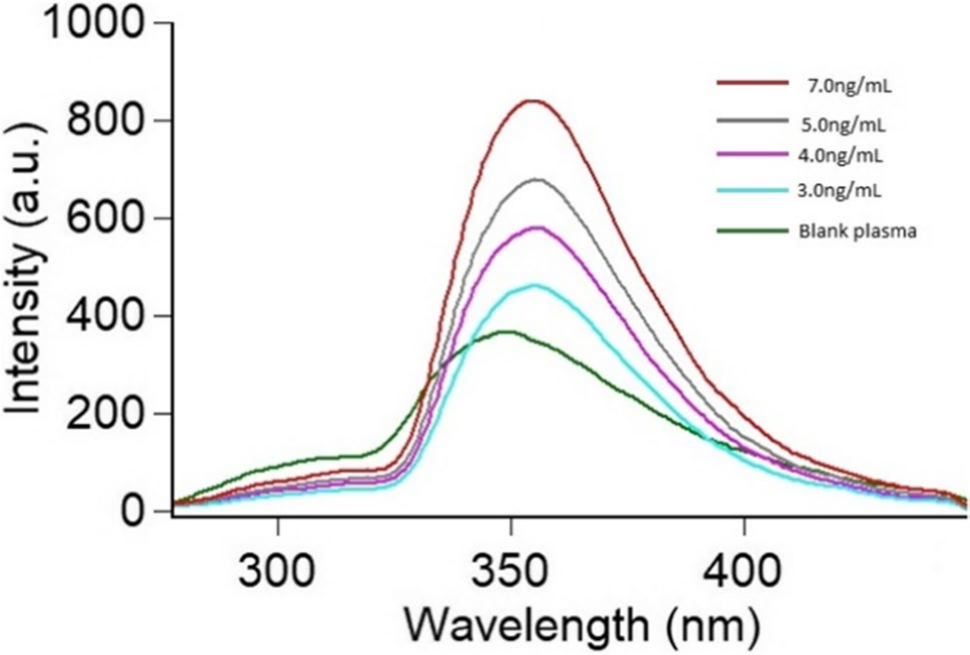
Application of the proposed spectrofluorimetric method for determination of CRS in spiked human plasma. All measurements were performed at 339 nm following excitation at 231 nm

Table 5 and Fig. 5 shows CRS determination in plasma samples in the range of 3.0 - 7.0 ng mL^⁻1^. The statistical analysis show that acceptable recovery percentages were reached between actual and estimated concentrations. Interference caused by plasma endogenous components was investigated using blank plasma samples, and the results at the wavelength of measurement revealed the minimization of such interference.

**Table 5:** Application of the suggested method to the determination of CRS in spiked human plasma

Drug	Assay results		
CRS	Amount taken (ng mL^-1^)	Amount found (ng mL^-1^)	Percent found
	3.00	2.95	98.38
	4.00	4.14	103.45
	5.00	4.89	97.80
	7.00	7.02	100.29
Mean ± S.D	99.98 ± 2.54		
% RSD	2.54		
% Error	1.27

### Evaluation of Method Greenness and Whiteness

#### Analytical Eco-Scale

Analytical Eco-Scale is classified as a semi-quantitative greenness tool Since it does not provide detailed data on the investigated approach. It is mainly utilized in laboratory practice and education purposes. The concept uses hazard pictograms to determine penalty points for instruments and reagents. The overall eco score = 100-the total number of penalty points [23], as shown in Table 6. The total Eco-score attained with the suggested strategy was 97 for raw material and capsules, and 86 for plasma application. According to the analytical eco-scale, methods showing total penalty points more than 75 are excellent green. Hence, the adopted approach is considered excellent green.

**Table 6:** Analytical eco-scale score for the suggested spectrofluorimetric method

Item	Penalty points in different matrix	
	Pure form and tablet	Plasma
Reagents: distilled water (diluting solvent)	0	0
Ethyl acetate (in plasma)	-	4
Acetonitrile (in plasma)	-	4
Instrument: spectrofluorimeter	0	0
Waste (1-10 mL)	3	3
Treatment: no treatment	3	3
Occupational hazard (no vapors or gases)	0	0
Total penalty points	**Ʃ6**	**Ʃ14**
Analytical eco-scale score	**94**	**86**

#### GAPI: Green Analytical Procedure Index

**Fig. 6:**
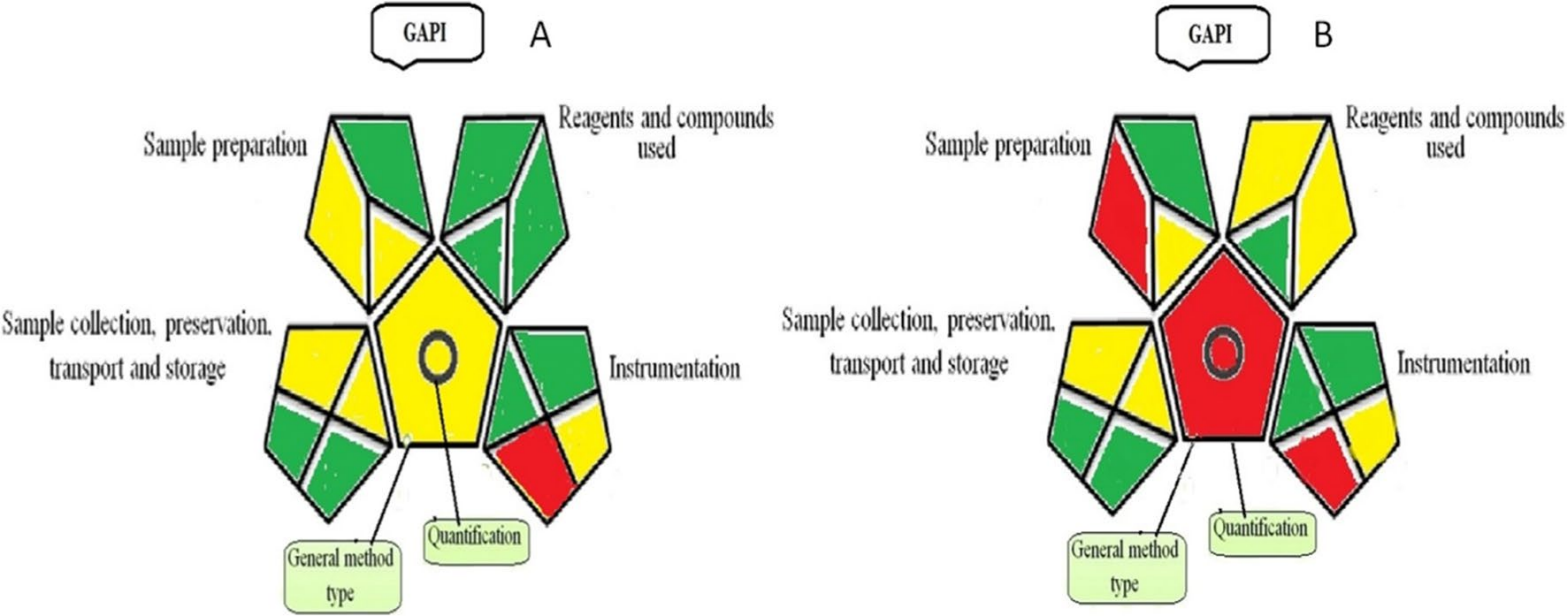
The green assessment profile for the suggested spectrofluorimetric method, using GAPI tool (**A**) for pure form and tablet & (**B**) for plasma

GAPI is a diagram designed to assess the overall greenness of a method. The diagram has fifteen sections correspond to five pentagrams, each of which addresses a different aspect of reagents, sample preparation, and chemicals used, apparatus, sample collection, and method type [24]. The suggested spectrofluorimetric approach met the GAPI criteria, as indicated in Table 7. As shown in Fig. 6A and B, each pentagram is divided into smaller portions that correspond to the stages of the pentagram title. The proposed method can be applied to qualification and quantification processes.

**Table 7:** Green Analytical Procedure Index (GAPI) parameters for the suggested spectrofluorimetric method for CRS

Category	Description	
	In pure form and tablet	In plasma
Sample preparation		
Collection (1)	on-line	on-line
Preservation (2)	None	None
Transport (3)	None	None
Storage (4)	normal condition	normal condition
Type of method: direct or indirect (5)	indirect (simple preparation(filtration)	Extraction required
Scale of extraction (6)	microextraction	microextraction
Solvents/reagents used (7)	green solvents/reagents	Non-green solvents/reagents
Additional treatments (8)	None	None
Reagent amount (9)	< 10 ml	< 10 ml
Health hazard (10)	slightly toxic	Moderately toxic
Safety hazard (11)	Safe	special hazard
Instrumentation		
Energy (12)	≤ 1.0 kWh per sample	≤ 1.0 kWh per sample
Occupational hazard (13)	hermetic sealing of the analytical process	hermetic sealing of the analytical process
Waste (14)	10 ml	10 ml
Waste treatment (15)	No treatment	No treatment
Quantification	Yes	Yes

#### AGREE: Analytical Greenness Metric Approach

**Fig. 7:**
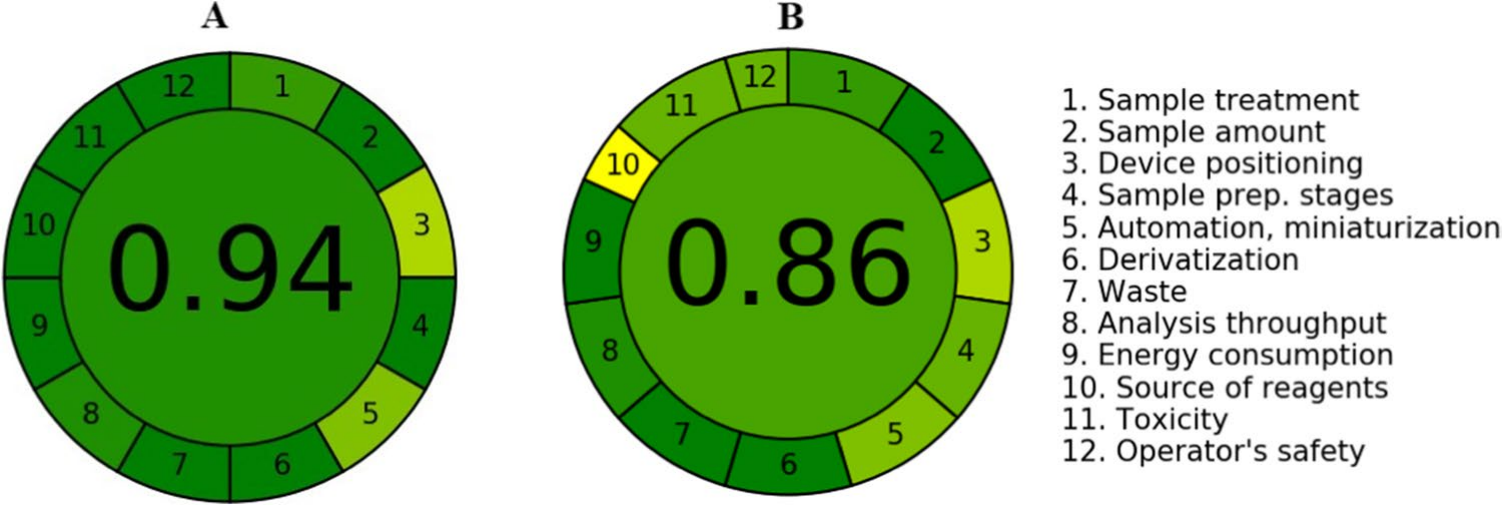
The green assessment profile for the suggested spectrofluorimetric method, using AGREE tool (**A**) for pure form and tablet & (**B**) for plasma

AGREE method consists of 12 evaluation criteria derived from the 12 green analytical chemistry principles and converted to a 0-1 scale. The final score is calculated using a specific piece of software in accordance with the 12 criteria [25]. When the estimated values approach one, the process becomes more environmentally friendly (Fig. 7 A and B).

#### Evaluation of Method Whiteness

The RGB algorithm, a metric tool of white analytical chemistry (WAC), was utilized to analyze the whiteness of the proposed method. Three factors are considered by WAC: analytical validity, economic feasibility, and ecological safety. Hence, this metric system is beneficial for determining the sustainability of an analytical procedure. Different colors correspond to each attribute, where red stands for analytical performance (R), green for environmental green impact (G), that is related to the green analytical chemistry derived 12 principles (GAC), and blue (B) for economic efficiency, productivity and practical applicability [26].

The whiteness of analytical methodology is represented by an end result ranging from white to black, based on the respective provisions of the three characteristics stated and the method's performance with regard to each aspect. The suggested strategy achieved 88.80% as an overall whiteness score (Table 8.), confirming the method's well balance and sustainability.

**Table 8.**
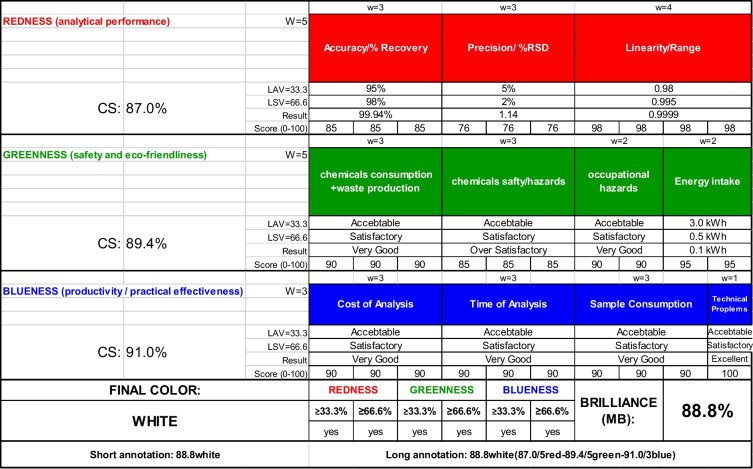
Whiteness assessment of the suggested spectrofluorimetric method for CRS applying RGB algorithm

## Conclusion

The flavonoid chrysin was estimated utilizing the first, ultra-fast, direct, reliable, and simple, validated spectrofluorimetric method. Chrysin was determined successfully utilizing the proposed technique in chrysin capsules and spiked human plasma samples. The methodology offered a high level of sensitivity, was significantly less expensive compared to HPLC-MS approaches, and entailed faster, simpler operations. The proposed approach received 97 total penalty points on the analytical eco-scale, 0.94 AGREE score, and 88.80 total whiteness score demonstrating alignment with the 12 principles of green analysis. Moreover, a total whiteness score of 88.80% was attained, highlighting the method sustainability and well balance.

## Data Availability

The raw data (including the spectrofluorimetric measurements and statistical analysis) that support the findings of this study are available upon request. dr.heba.samir@mans.edu.eg.
